# Mammary Analogue Secretory Carcinoma of the Parotid Gland: A Third World Country Perspective—A Case Series

**DOI:** 10.1155/2015/697254

**Published:** 2015-12-13

**Authors:** Huzaifah Salat, Ramiz Mumtaz, Mubasher Ikram, Nasir Ud Din

**Affiliations:** ^1^Aga Khan University Medical College, Karachi 74800, Pakistan; ^2^Department of Otolaryngology, Aga Khan University, Karachi 74800, Pakistan; ^3^Department of Pathology & Microbiology, Aga Khan University, Karachi 74800, Pakistan

## Abstract

Mammary analogue secretory carcinoma (MASC) is a recently described pathological entity in major salivary glands, which was first described by Skálová et al. in 2010. Since then only a limited number of case reports/series have been published describing this tumor with the majority of them discussing the genetic and cytoarchitectural aspect of this tumor. Keeping this in view with the lack of clinical correlation with regard to this tumor, we present our approach to management of two such cases which, according to the best of our knowledge, are the first 2 cases presenting in the South Asian continent. Both patients were diagnosed and managed at Aga Khan University Hospital, Karachi, Pakistan.

## 1. Introduction

Mammary analogue secretory carcinoma (MASC) is a recently described pathological entity in major salivary glands, with the parotid being the most common location. This salivary gland tumor has histopathological features synonymous of both salivary acinic cell carcinoma (AciCC) and low grade cystadenocarcinoma while at the same time strongly displaying significant similarities to breast secretory carcinoma [1]. Microscopically it comprises solid and microcystic areas, with microcysts filled with colloid-like PAS positive material and appearance with low grade nuclei and granular cytoplasm [2].

WHO classification of salivary gland neoplasms is complex and consists of 10 benign and 23 malignant variants [3]. Regardless, salivary gland neoplasms are not common as literature reveals an epidemiologic prevalence of 0.4–13.5 cases per 100,000 people. Among the salivary gland tumors, parotid gland tumors are rare in entity themselves and account for 12% of oropharyngeal cancers or 0–3% of all cancers affecting the body [4]. In 2010, the first reported case series by Skálová et al. consisted of 16 cases out of which 13 were of parotid origin and 3 originated from minor salivary glands [5]. Henceforth, we present 2 cases of MASC of the parotid gland which to our knowledge are the first two cases in the South Asian territory. Both patients were diagnosed and managed at Aga Khan University Hospital, Karachi, Pakistan.

## 2. Case 1

A 60-year-old man, resident of Sukkur, Sindh, and an occasional alcoholic, presented to the ENT clinic with a seven-month history of swelling present on the right parotid area which was initially small but increasing in size gradually. He had no other associated symptoms. Past medical history and family history were not significant. On examination there was a 3 × 3 cm firm swelling present at the right parotid area, which was mobile in all planes. Overlying skin was not involved and no neck nodes were palpable. Rest of the ENT examination was unremarkable.

**Figure 1:**
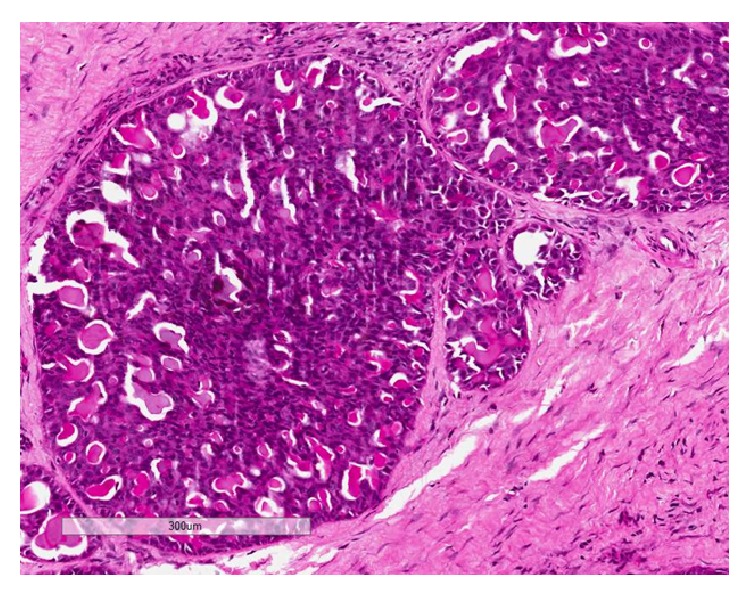
MASC displaying microcystic pattern of growth and these spaces are filled with pink colloid-like material (H&E, 200x magnification).

The FNAC of the swelling was done which showed epithelial neoplasm possibly of salivary gland origin. So with the provisional diagnosis of suspected malignancy of right parotid gland he underwent a right parotidectomy. Intraoperatively, the mass was 4-5 cm, hard lesion in the superficial lobe which was removed with clear margins while preserving the facial nerve. The final histopathology result revealed mammary analogue secretory carcinoma which was reconfirmed by four consultant histopathologists (Figure 1).

The case was then discussed in multidisciplinary tumor (MDT) meeting and it was recommended that a neck dissection should be carried out, followed by radiation therapy which was total dose of 66 Gy in 33 cycles. Subsequently, he underwent modified radical neck dissection on the same side followed by radiation therapy.

On follow-up after 1 month and 7 months, respectively, the patient was stable and was allowed to go back to his hometown.

## 3. Case 2

A 42-year-old male, a resident of Jacobabad, Sindh, with no known comorbid conditions and no history of addiction, visited our clinic in February 2013 with a 1-year history of swelling present in the left parotid area. He was operated on outside our institute in 2012 with the preliminary diagnosis of pleomorphic adenoma of parotid gland, but the final histopathology report revealed that there was a suspicion of malignancy with involved margins coming back positive as well. Past medical history and family history were unremarkable for this patient as well.

When we examined the mass it was 2 × 2 cm left preauricular swelling which was tender, firm, and mobile. We repeated his MRI scan which showed residual mass of left parotid gland extending up to left mastoid with no nodal involvement. Subsequently, he was planned for left total parotidectomy. Intraoperatively, the lesion was found in the tail of parotid, and thus revision left total parotidectomy was done. Neck dissection was not done in this case because there were no suspicious nodes detected on radiologic scan.

**Figure 2:**
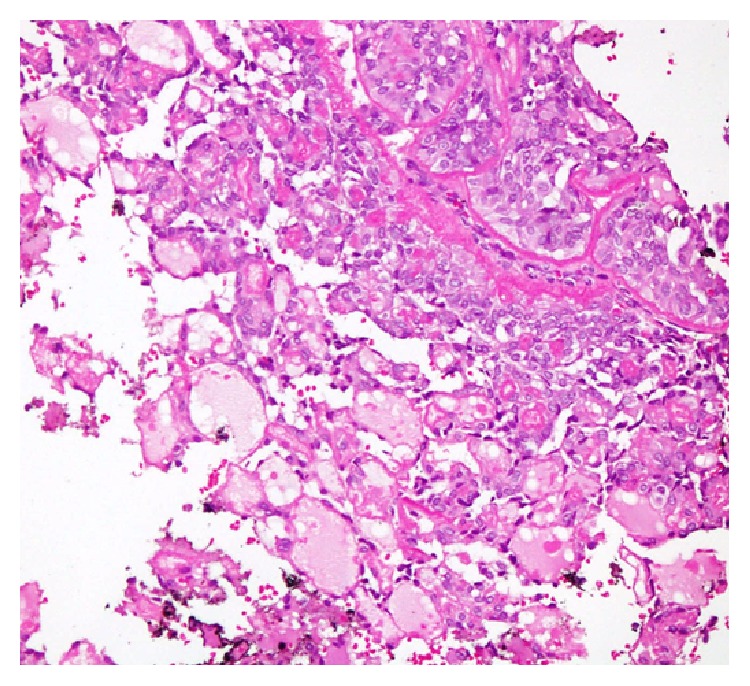
MASC displaying microcystic pattern of growth with the spaces showing pink colloid-like material.

Final histopathology report showed MASC (Figure 2). The case was then discussed in MDT meeting, and it was decided that the patient should receive adjuvant radiotherapy with total dose of 66 Gy.

On follow-up after 1 month and 6 months, respectively, the patient was deemed stable and was advised to come for yearly follow-up.

## 4. Discussion

In 2010, a case series comprising 16 cases was published by Skálová et al., in which a new salivary gland tumor was described and named MASC [5]. Since then, to the best of our knowledge, 92 cases [3] have been published describing this hitherto undescribed tumor, with the majority of the articles being review articles or articles discussing the genetic and cytoarchitectural aspect of this tumor. Keeping this in perspective with the lack of clinical correlation with regard to this tumor, we have highlighted our experience of management in these cases which according to our knowledge are the first two cases reported in South Asia.

Due to the considerable overlap of histological features of this tumor with other aforementioned malignancies, FNA aspirates are sometimes notable to give a conclusive answer. This was true with regard to our patient as well, as our initial FNAC result suggested a tumor of epithelial origin. However, among the 12 FNA diagnosed cases of MASC published [3], only one [6] received an initial diagnosis of MASC. The question of whether surgical biopsy is the best method of diagnosis still remains unanswered. As far as diagnosis is concerned in developing countries, extensive immunohistochemical analysis is not possible. Firstly, it is not easily available and secondly because health care in developing countries like Pakistan is through one's own pockets with no federal government or insurance companies to support, immunohistochemical analysis remains to be an academician dream. Postoperative radiotherapy (PORT) is usually done for close (<5 mm) margins/incomplete resection, perineural invasion, and all tumors greater than T2 size, whereas in cases of suspicion of low grade malignancy radical surgical resection is usually the standard protocol [7].

Hence, to bridge the gap between diagnostics and interventions, ancillary studies are being carried out to effectively diagnose MASC using various modalities. Genetic studies suggest that these tumors possess a chromosomal translocation t(12;15)-(p13;q25) analogous to breast carcinoma, resulting in a fusion oncogene ETV6-NTRK3 [8]. This fusion results in the activation of tyrosine kinase which further potentiates the activation of Ras-MAP mitogen pathway and phosphatidylinositol 3-kinase-AKT pathway [9, 10]. The significance of this translocation is that this was the exact translocation first detected in infantile fibrosarcoma and congenital mesoblastic nephroma [11, 12]. In addition, similar translocations have also been consistent findings in secretory carcinoma of breast, hence the name secretory analogue carcinoma [5].

**Figure 3:**
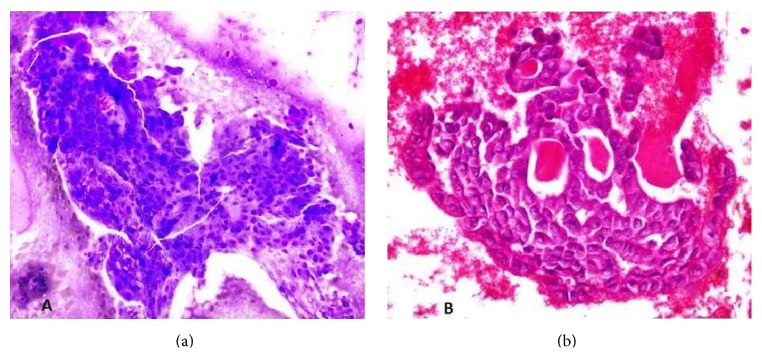
Cytology smears of FNAC ((a) Diff-Quick and (b) H&E stain) show a low grade epithelial tumor with round to oval nuclei. Eosinophilic secretions are seen in H&E image (b).

Keeping in mind the complexity and heterogeneity of presentations of salivary gland tumors and to effectively diagnose our cases, we used fine needle aspiration cytology and immunohistochemistry markers such as S100, Cytokeratin 19, EMA (epithelial membrane antigen), and DOG 1 stain. Both our cases on histology specimens confirmed low grade MASC tumor as they grew in a microcystic pattern, with eosinophilic secretions noted in the luminal spaces along with uniform nuclei (Figure 3). We ruled out acinic cell carcinoma (AciCC) on the basis of DOG1 stain which is negative in MASC and the fact that AciCC should at least demonstrate focal basophilic cytoplasmic granules and macronuclei [5]. We also ruled out low grade mucoepidermoid carcinoma (MEC) as our cases did not display the abundant epidermoid cells and mucinous cells frequently seen in MEC [13]. S100 and EMA markers are positive in MASC, whereas DOG1 stain is positive in majority of cases of AciCC [5]. These modalities were enough to come to a clear diagnosis of MASC and we recommend use of these markers along with histopathology to come to a diagnosis in resource limited countries, where extensive histochemical studies cannot be performed.

Treatment modalities mentioned in literature include details of 86 patients. 26% of the patients underwent neck dissections and 20% of patients were given postoperative radiotherapy (PORT), while 2% of the patients received PORT with chemotherapy. No reported patients were given radiotherapy before surgery. Due to the limited data available with regard to treatment protocols, surgeons may currently consider neck dissection as the safest intervention, especially since we cannot conclude whether MASC comes under the category of low grade malignant salivary tumors or not.

## 5. Conclusion

Published data remains unclear as to which modality is more superior over the other. Overwhelming amount of research is being done on the cytochemical aspects of MASC rather than on its diagnosis and management. Further research needs to be done in this area to understand the prognostic significance of MASC.

In South Asia, where there is a dearth of surgeons and lack of available extensive immunohistochemical diagnostic modalities, especially in the developing countries, we advise close clinical and surgical correlation with respect to the management of MASC via multidisciplinary tumor board meetings and strongly suggest that MASC be kept in the differential diagnosis in tumors arising from salivary glands.
